# The Exocytosis Associated SNAP25-Type Protein, SlSNAP33, Increases Salt Stress Tolerance by Modulating Endocytosis in Tomato

**DOI:** 10.3390/plants10071322

**Published:** 2021-06-29

**Authors:** Josselyn Salinas-Cornejo, José Madrid-Espinoza, Isabel Verdugo, Jorge Pérez-Díaz, Alex San Martín-Davison, Lorena Norambuena, Simón Ruiz-Lara

**Affiliations:** 1 Laboratorio de Genómica Funcional, Instituto de Ciencias Biológicas, Universidad de Talca, Talca 3460000, Chile; josalinas@utalca.cl (J.S.-C.); jmadrid@utalca.cl (J.M.-E.); iverdugo@utalca.cl (I.V.); jorgelperezd@gmail.com (J.P.-D.); alexsanmartin@utalca.cl (A.S.M.-D.); 2 Facultad de Ciencias, Universidad de Chile, Santiago, Ñuñoa 7750000, Chile; lnorambuena@uchile.cl

**Keywords:** SNARE, SNAP33, *S. lycopersicum*, endocytosis, salt stress, sodium compartmentalization

## Abstract

In plants, vesicular trafficking is crucial for the response and survival to environmental challenges. The active trafficking of vesicles is essential to maintain cell homeostasis during salt stress. Soluble N-ethylmaleimide-sensitive factor attachment protein receptors (SNAREs) are regulatory proteins of vesicular trafficking. They mediate membrane fusion and guarantee cargo delivery to the correct cellular compartments. SNAREs from the Qbc subfamily are the best-characterized plasma membrane SNAREs, where they control exocytosis during cell division and defense response. The *Solanum lycopersicum* gene *SlSNAP33.2* encodes a Qbc-SNARE protein and is induced under salt stress conditions. *Sl*SNAP33.2 localizes on the plasma membrane of root cells of *Arabidopsis thaliana*. In order to study its role in endocytosis and salt stress response, we overexpressed the *SlSNAP33.2* cDNA in a tomato cultivar. Constitutive overexpression promoted endocytosis along with the accumulation of sodium (Na^+^) in the vacuoles. It also protected the plant from cell damage by decreasing the accumulation of hydrogen peroxide (H_2_O_2_) in the cytoplasm of stressed root cells. Subsequently, the higher level of *SlSNAP33.2* conferred tolerance to salt stress in tomato plants. The analysis of physiological and biochemical parameters such as relative water content, the efficiency of the photosystem II, performance index, chlorophyll, and MDA contents showed that tomato plants overexpressing *SlSNAP33.2* displayed a better performance under salt stress than wild type plants. These results reveal a role for *Sl*SNAP33.2 in the endocytosis pathway involved in plant response to salt stress. This research shows that *SlSNAP33.2* can be an effective tool for the genetic improvement of crop plants.

## 1. Introduction

In eukaryotic cells, a vesicular trafficking system allows communication between their internal compartments and neighboring cells. At the plant scale, this vesicular traffic is essential for growth, development, and responses to environmental challenges [1,2]. The specificity of material transfer between compartments through vesicular trafficking is ensured by selective incorporation of cargo during vesicle formation on the one hand and a specific fusion of the vesicle to the acceptor compartment membrane on the other hand [3,4]. Soluble N-ethylmaleimide-sensitive factor attachment protein receptors (SNAREs) form a superfamily of proteins remarkably conserved from yeast to mammals that ensure the specificity of the membrane fusion [2,3,4]. Vesicle fusion is driven by the physical interaction between partners SNARE anchored at both the vesicle membrane and the membrane of the target compartment [5]. Interactions between SNARE domains of SNARE proteins ensure the docking of the two membranes and generates a force that promotes the fusion of the vesicle. Most commonly, SNARE domains interaction form a tetrameric complex that has a four-helical bundle structure: one helix is provided by a v-SNARE located on the vesicle membrane (R-SNARE) while the three other helices correspond to three independent t-SNAREs on the target membrane (SNAREQa, Qb, and Qc, respectively) [5,6]. However, membrane fusion is also possible with a SNARE complex formed by only three proteins instead of four. This occurs when a Q-SNARE has two SNARE domains in its polypeptide chain, as is the case for the Qbc-SNAREs. This feature makes those Qbc-SNARE remarkable. One of them is the Synaptosome-associated protein 25 (SNAP25) involved in the fusion of vesicles with the plasma membrane. Another structural feature unique to Qbc-SNAREs is their integration into the membrane; while most SNAREs display a transmembrane domain, SNAP25-type proteins are attached to the membrane by a lipidic group added by posttranslational modification [7], further suggesting important differences in the molecular mechanism of Qbc-SNAREs-mediated membrane fusion compared to other SNARES.

The SNAP25-type proteins were discovered in mammals (SNAP23, SNAP25, SNAP29, and SNAP47), and homologs were later identified in yeast and plants [3]. In all those organisms, SNAP25-type proteins fulfill critical functions in the vesicular trafficking associated with the exocytic pathway [3,8]. In plants, the first characterized SNAP25-type protein was *At*SNAP33 in *Arabidopsis*. *At*SNAP33 participates in the formation of the phragmoplast during late cytokinesis in interaction with both KNOLLE (Qa-SNARE SYP111) and *At*VAMP721 (R-SNARE) [3,9]. Accordingly, the expression of *CkSNAP33* of *Cynanchum komarovii* in *A. thaliana* results in the increase in both the root cell number and leaf size associated with a dysregulation of the cytokinesis [10,11]. In *A. thaliana,* other SNAP25-type proteins, namely *AtSNAP29* and *AtSNAP30,* have been proposed to be involved in the cytokinesis process in specific organs such as the flower and the root [3].

SNAP25-type proteins are also involved in response to biotic and abiotic stress. In particular, *At*SNAP33 interacts with *At*PEN1/*At*SYP121 and *At*VAMP721/722 SNARE proteins and participates in the innate immune response by promoting the release of vesicles that carry defense cargo at fungal penetration sites [12,13,14]. Similarly, ectopic expression of both *CkSNAP33* and its homologous *GhSNAP33* from *Gossypium hirsutum* enhance the resistance of Arabidopsis plants to *Verticillium dahliae* [10,15]. In the same line of thought, *StSNAP33* in *Solanum tuberosum* plays a role in the defense response to *Pseudomonas syringae* pv. maculicola and *Phytophthora infestans* of potato plants [16]. Altogether, these results suggest that the orthologs of *AtSNAP33* conserved their functions as a component of a trimeric SNARE complex during plant response to biotic stress in dicots and monocots [3,17].

Three Qbc-SNAREs have been identified in the genomes of *Solanum lycopersicum* and its relative wild species (*S. chilense*, *S. pimpinellifolium*, *S. habrochaites*, and *S. pennellii*), namely *SlSNAP33.1*, *SlSNAP33.2*, and *SlSNAP30* [18]. The transcripts accumulation of the three *AtSNAP33* orthologs *SlSNAP33.2, SchSNAP33.2*, and *GsSNAP33* from *Glycine soja* increase during salt stress [18,19], suggest that SNAP33 orthologues play a role in the plant adaptation to abiotic stress.

Vesicular trafficking plays an important role in plant development and growth, as well as in abiotic stress tolerance. Leshem et al. (2007) [20] reported that salt stress induces bulk-flow endocytosis in Arabidopsis roots, which has been confirmed in other studies [21,22，^23^]. Such endocytosis activation promotes a rapid increase in vacuolar volume, accumulation of sodium into the vacuole of root cells [24,25], and internalization of the plasma membrane proteins into endosomal compartments [26]. This suggests that proteins involved in driving the endocytic trafficking play a role in salt stress adaptative response.

As mentioned above, SNAP33 participates in exocytic trafficking [3,27]. Accordingly, its overexpression induces an increase in exocytic vesicular trafficking, but its role in endocytosis has not been evaluated. In this work, we characterized *Sl*SNAP33.2 as a SNARE protein on the plasma membrane with a role in endocytosis associated with plant response to salt stress. *Sl*SNAP33.2 overexpression in *S. lycopersicum* promoted endocytosis and increases the compartmentalization of sodium into the vacuoles during salt stress. The overexpression also reduced the concentration of hydrogen peroxide inside root cells, protecting them from ROS-dependent salt-induced damages. More importantly, the overexpression of *SlSNAP33.2* increases the tolerance to salt stress of tomato plants. Altogether, these results place the SNARE *SlSNAP33.2* as an important player in the endocytic vesicle trafficking with a role in the adaptative mechanisms that allow the plant to cope with salt stress.

## 2. Results

### 2.1. SlSNAP33.2 Localizes on the Plasma Membrane

**Figure 1:**
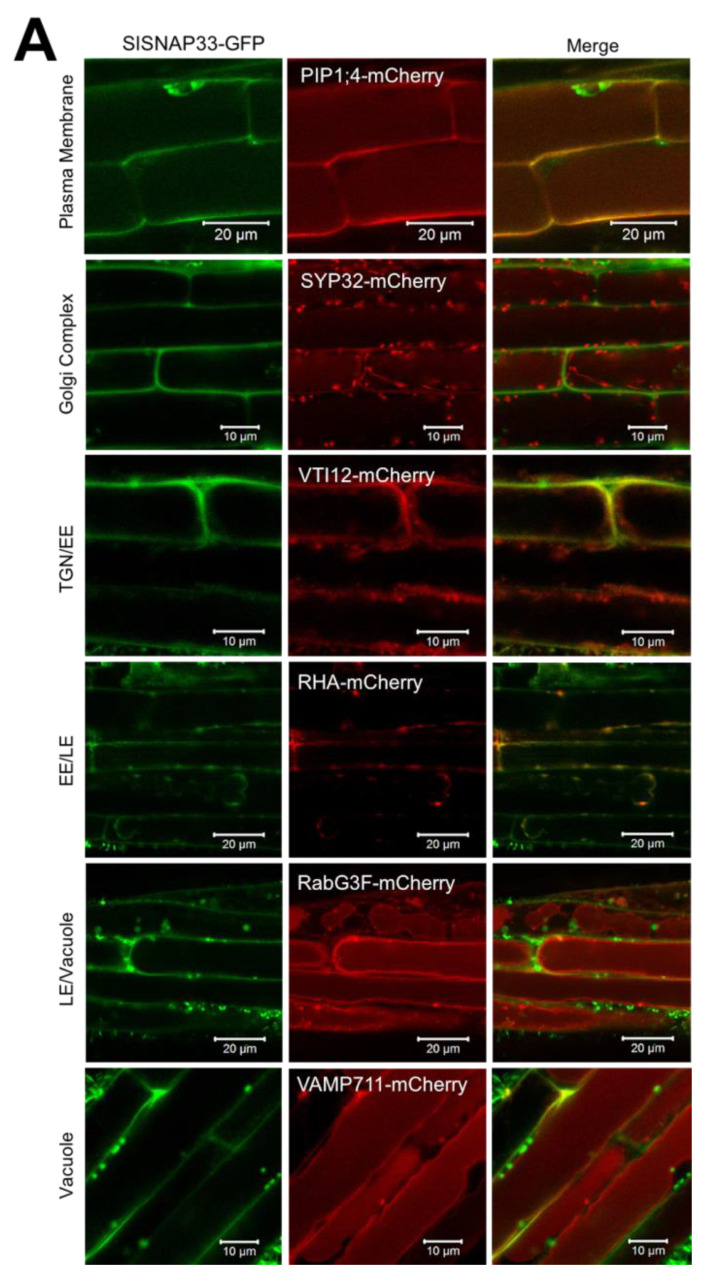
Subcellular localization of SlSNAP33.2 in root cells of *A. thaliana*. SlSNAP33.2-GFP was transiently expressed in Arabidopsis plant lines expressing mCherry fusion marker. GFP fluorescence is visualized in green while mCherry is in red on confocal images. Lines that express fluorescent mCherry Subcellular markers: PIP1;4-mCherry, SYP32-mCherry, VTI12-mCherry, RHA-mCherry, RabG3F-mCherry, and VAMP711-mCherry, with specific locations as indicated in the figure. TGN: Trans-Golgi Network; EE: Early endosome; LE: Late endosome.

SNARE genes have been identified in the genome of the commercial tomato *S. lycopersicum*. Salinas-Cornejo and colleagues [18] identified 63 putative SNARE genes that segregate into five phylogenetic clades. *Sl*SNAP33.2 is predicted to localize on the plasma membrane as its paralogs do [18]. To confirm this location, the *Sl*SNAP33.2 CDS was cloned and fused to the GFP in an expression vector (*p35S::SlSNAP33.2::GFP*). Previously characterized *A. thaliana* plants expressing fluorescent genetic markers specific for Golgi, Trans Golgi network, Late endosome, tonoplast, and plasma membrane, respectively, were used to identify the subcellular location of *Sl*SNAP33.2::GFP [28]. Root cells from those plants were transiently transformed with p*Sl*SNAP33.2::GFP using AGROBEST technique [29], and the transformed cells were analyzed under the confocal microscope. As expected, the GFP signal from control cells expressing the GFP was distributed all over the cell (Figure S1). In contrast, the signal observed in cells from all Arabidopsis marker lines transformed with *pSlSNAP33.2::GFP* was restricted to a structure surrounding the cell, typical of PM-localized proteins (Figure 1). Indeed, the *Sl*SNAP33.2::GFP signal displayed good overlap with the PM-localized *At*PIP1;4::mCherry signal (Figure 1). *Sl*SNAP33.2::GFP did not localize at the Golgi complex, and no overlap was observed between the GFP and mCherry signals in cells expressing the Golgi-localized probe *At*SYP32-mCherry. No signal merging was observed between GFP and mCherry in cells expressing either the Trans-Golgi Network (TGN) markers *At*VTI12-mCherry or *At*PVCs *At*RHA-mCherry or the late endosome and vacuole markers *At*RabG3f-mCherry and *At*VAMP711-mCherry, respectively.

### 2.2. Constitutive Expression of SlSNAP33.2 Enhances Tolerance to Salt Stress in Tomato

Although previous analyzes showed that the overexpression of *SNAP33* in different plant species confers tolerance to abiotic stress [19], no such data is available in plants of agronomic interest, such as tomato plants. Strong induction of *SlSNAP33.2* transcription has been observed in response to salt stress in different species of tomato [18], suggesting a role for *SlSNAP33.2* in the adaptative response. To test this hypothesis, we evaluated the effect of the overexpression of *SlSNAP33.* in *S. lycopersicum*. We cloned the CDS of *SlSNAP33.2* downstream of the constitutive CaMV35S promoter in the pBI121 vector (*p35S::SlSNAP33*). Tomato plants were transfected, and transgenic plants were selected. Tomato plants transformed with the empty vector (*pBI121-empty*) were used as a control.

Out of sixteen selected independent *p-35S::SlSNAP33* transgenic lines, five were analyzed (L1, L3, L5, L8, and L12). The *SlSNAP33.2* expression level was evaluated using qRT-PCR. The expression level of *SlSNAP33.2* in L3 was 22 times higher than in the wild-type plants, while it was about 10 times higher in the lines L1, L5, and L12 (Figure S2). Based on these analyses, lines L3 and L5 were chosen for further characterization.

**Figure 2:**
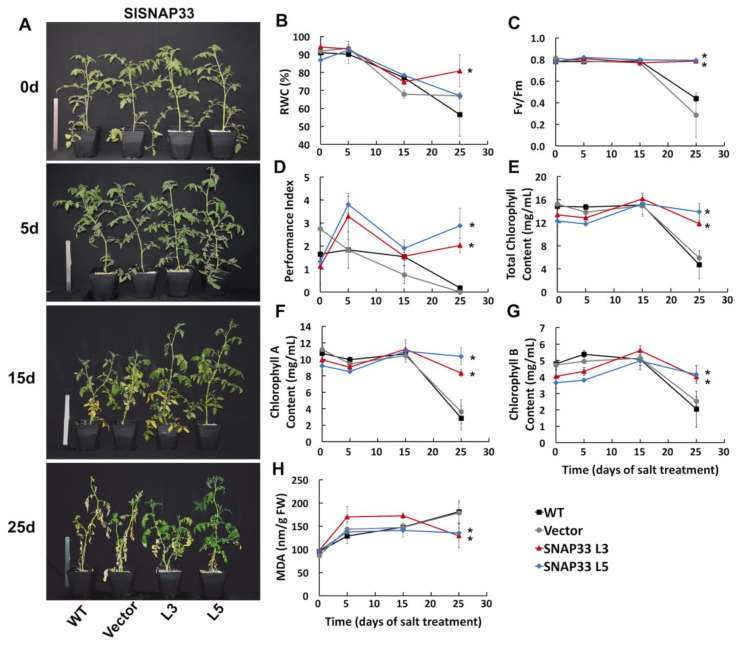
Physiological parameters of *SlSNAP33.2* overexpressor tomato lines subjected to saline stress. (**A**) Images of 10-11-week-old tomato plants after 0, 5, 15, and 25 days of salt stress treatment (300 mM NaCl); (**B**–**H**) Physiological parameters were measured on *pBI121-35S::SlSNAP33* as well as on *pBI121-empty* and wild type control plants on the time points showed in (**A**). (**B**) Leaf Relative Water Content (RWC); (**C**) Maximal photochemical efficiency of PSII (Fv/Fm); (**D**) Index performance (PI); (**E**) Total chlorophyll content (mg/mL); (**F**) Chlorophyll A content (mg/mL0); (**G**) Chlorophyll B content (mg/mL); (**H**) Malondialdehyde (MDA) content (mg/g FW). FW: Fresh Weight. Mean ± standard error. Asterisks at the end of each line indicate a statistically significant difference (*p* < 0.05) between WT and transgenic lines on day 25 according to a one-way ANOVA test.

To test whether the overexpression of *SlSNAP33.2* affects tomato plants salt tolerance, salt stress assays were performed on 10 to 11-week-old plants. Tomato plants were irrigated with 300 mM NaCl at 3-day intervals for 25 days. Five days after starting the salt stress, the comparison of control *pBI121-empty* plant lines with L3 and L5 *p-35S::SlSNAP33* plants lines showed no apparent phenotype, suggesting that the level of *SlSNAP33.2* expression did not affect development or growth in control conditions. After ten days, all lines started showing yellow leaves as symptoms of NaCl-induced chlorosis. At this time, L3 and L5 plant lines started showing a phenotype characterized by a bigger size than WT and *pBI121-empty* control plants (Figure 2A). The growth phenotype developed with time and was very obvious after 25 days of salt stress. At this time, symptoms of chlorosis also coincided with the *SlSNAP33* expression level, with yellow leaves and loss of leaves tissues much more apparent in the WT and *pBI121-empty* lines than in *pBI121-35S::SlSNAP33* genotypes (Figure 2A). This set of data indicate that *SlSNAP33.2* overexpression somehow promotes salt stress tolerance.

Physiological and biochemical parameters were further measured in order to understand the physiological mechanism by which *SlSNAP33.2* confers salt tolerance to the overexpressing plants. The relative water content (RWC) decreased below 70% after 15 days of stress in the control plants, reaching an RWC of less than 50% at the end of the treatment (Figure 2B). In contrast, after 25 days of salt treatment, plants from the L3 line were able to maintain their relative water content stable, showing RWC decreased by only 20% (Figure 2B). Along with RWC, the maximum efficiency of PSII (Fv/Fm) (Figure 2C) and the performance index (PI) (Figure 2D) were measured in all plants subjected to salt stress. In control plants, these parameters displayed a rapid decrease after 15 days and until the end of the assay. In contrast, in L3 and L5 lines, PSII efficiency and PI remained stable throughout the salt treatment, with an Fv/Fm of 0.8 (Figure 2C) and PI between 2 and 3 (Figure 2D), indicating a better performance at a physiological level. Last, after 25 days of salt stress, L3 and L5 lines showed a higher chlorophyll content (Figure 2E–G) and lower MDA (Figure 2H) compared to the control plants. Altogether, these results indicate that the overexpression of *SlSNAP33.2* in tomato plants confers tolerance to high salinity by improving physiological and biochemical parameters that are affected when plants are under salt stress conditions.

### 2.3. SlSNAP33.2 Enhances Endocytic Vesicular Trafficking in Salt-Stressed Tomato Root Cells

**Figure 3:**
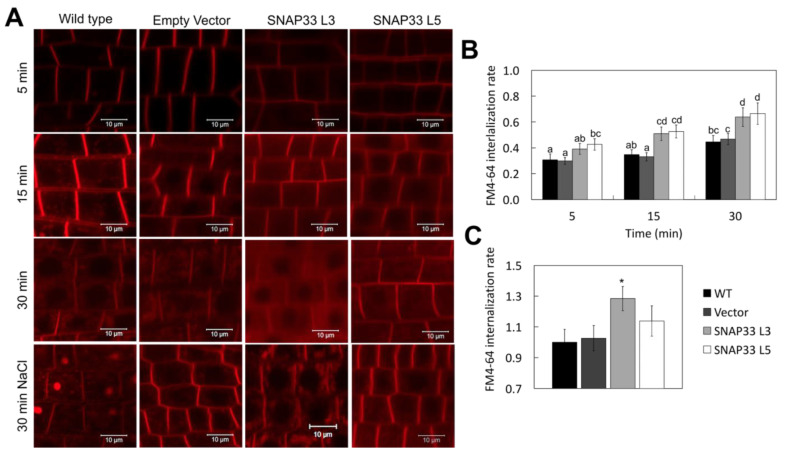
Endocytic trafficking of FM4-64 in tomato lines. (**A**) Representative images of the FM4-64 internalization monitored in different time points in control (WT and *pBI121-empty*) and *SlSNAP33.2* overexpressor tomato lines under normal conditions and under-treatment of 200 mM NaCl. Scale bar: 10 µm. (**B**) Quantification of the internalization rate of FM4-64 under normal conditions in tomato roots. Data are expressed as mean ± standard error; *n* = 25 cells. Different letters indicate significant differences (*p* < 0.05) according to a one-way ANOVA test. (**C**) Quantification of the FM4-64 internalization rate under 200 mM salt stress. The values are relative to the endocytic rate of WT under conditions of saline stress. Data are represented as means ± standard deviation. Asterisks indicate statistically significant differences (*p* < 0.05) according to a one-way ANOVA test.

*Sl*SNAP33.2 is associated with the plasma membrane (Figure 1), and to test the hypothesis that it plays a role in the control of the endocytic pathway [3], the internalization rate of the tracer FM4-64 in tomato root cells was analyzed. After binding to the plasma membrane, FM4-64 was internalized by bulk endocytosis, reaching endosomal compartments (Figure 3). Endocytosis proceeded in both control and *SlSNAP33.2* overexpressing plants, but at a different rate. Indeed, root cells of L3 and L5 accumulated higher amounts of the tracer a t = 15 min and t = 30 min after the addition of the FM4-64, respectively, compared with cells from control plants (20% increase in the internalization rate, see Figure 3A,B). This indicates that the higher level of SlSNAP33.2 promoted endocytic trafficking. Salt stress-induced endocytosis in control plants (WT and Vector, Figure 3A) as has been described previously [20,21,22，^23^]. Salt treatment induced a 30% higher rate of endocytosis on root cells from L3 compared to root cells from control plants (Figure 3A,C). In root cells from L5, it seemed to display a difference to control cells on the endocytosis rate; however, the difference was not statistically supported. The differences observed between L3 and L5 lines were coincident with the higher expression level of *SlSNAP33.2* (Figure S2), revealing a dose-response effect.

### 2.4. SlSNAP33.2 Reduces Production of H_2_O_2_ in Transgenic Tomato Root Cells during Salt Stress

**Figure 4:**
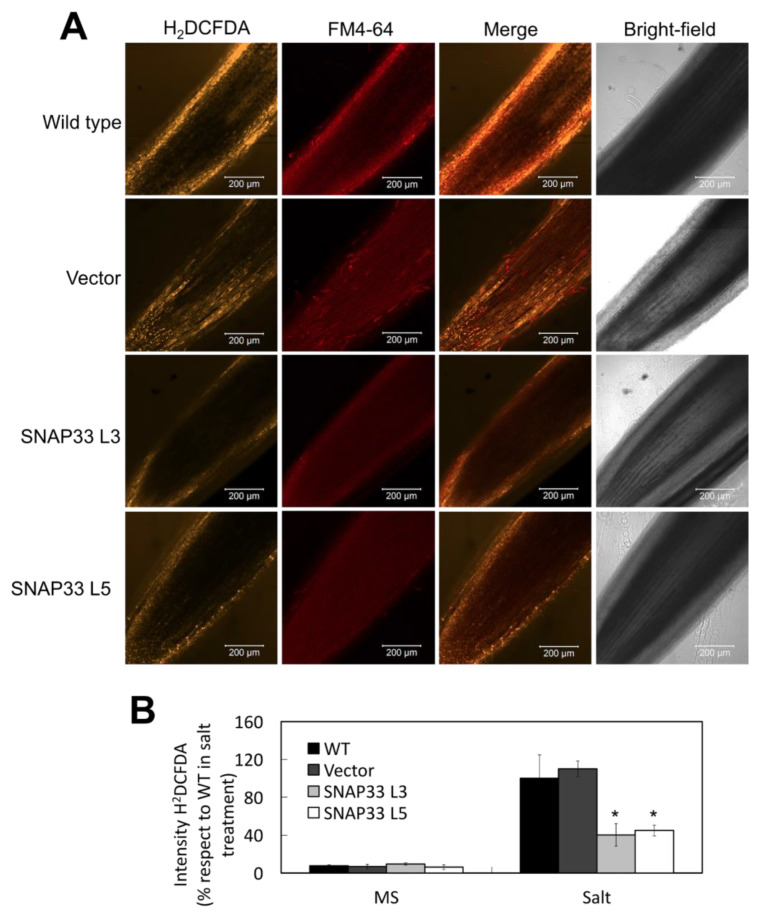
H_2_O_2_ production in tomato lines during saline stress. (**A**) Representative images of roots of control lines (WT and Vector) and *SlSNAP33.2* overexpressor tomato. Roots were treated with H_2_DCFDA (yellow signal) and FM4-64 (red signal) in the presence of 200 mM NaCl. Scale bar: 200 μm. (**B**) Quantification of the DCF fluorescence. The values are percentages relative to the intensity of the fluorescence evaluated in WT under saline stress treatment (100%). The data are represented as the percentage ± standard deviation. Asterisks indicate statistically significant differences (*p* < 0.05) according to a one-way ANOVA test.

To demonstrate whether the increase in tolerance to salt stress in *SlSNAP33.2* overexpressing tomato plants was related to the level of ROS production, H_2_O_2_ concentrations were determined in the roots of seedlings subjected to 200 mM NaCl using a fluorescent probe under the confocal microscope. The fluorescence of DCF was measured as the oxidation product of H_2_DCFDA when H_2_O_2_ is present. The level of DCF is an indicator of the amount of H_2_O_2_ in the tissue. There was no difference in H_2_O_2_ level between control and *SlSNAP33.2* overexpressor plants in control conditions (Figure S3). In comparison, after 60 min of salt treatment though (200 nM NaCl), control plants displayed higher levels of DCF than *p-35S::SlSNAP33.2* (Figure 4A). Note that under the same treatment, DCF increase was about 60% lower in L3 and L5 lines (Figure 4B). This indicates that the increment of *Sl*SNAP33.2 prevents ROS accumulation when plants were exposed to salt stress.

### 2.5. The Na^+^ Vacuolar Compartmentalization Capacity Was Increased in Transgenic Tomato Root Cells

**Figure 5:**
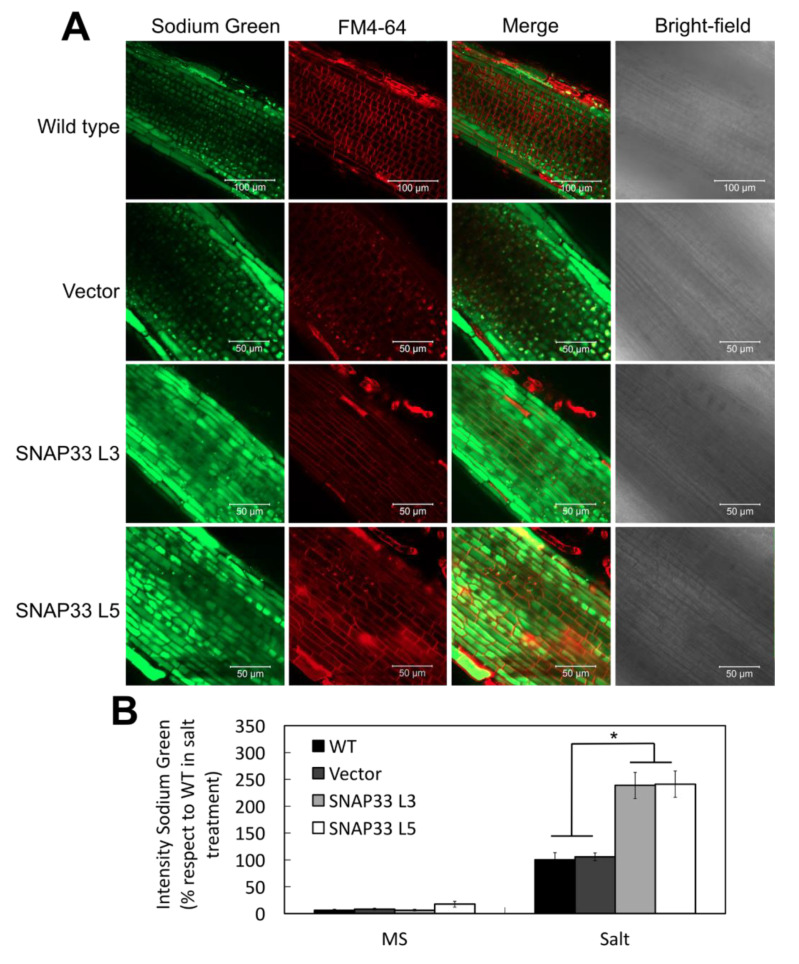
Identification of Na^+^ in tomato lines under salt stress. (**A**) Representative images of roots of control lines (WT and Vector) and *SlSNAP33.2* overexpressor tomato lines under salt stress. Roots were treated with Sodium-Green (green signal) and FM4-64 (red signal). Scale bar 50–100 μm. (**B**) Quantification of Sodium-Green fluorescence. The Na^+^ content on WT plants under salt stress was considered 100%. Data are represented as the percentage ± standard deviation. Asterisk indicates statistically significant differences (*p* < 0.05) according to a one-way ANOVA test.

Under salt stress, the excess of Na^+^ is sequestered into the vacuole allowing a relative salt tolerance. The induction of the endocytic rate promotes such Na^+^ compartmentalization [30]. Taking into account the phenotypes displayed by L3 and L5 lines, the Na^+^ content was monitored by using the Sodium-green indicator. Without salt stress, the intensity of Sodium-green fluorescence was at the detection limit level in all genotypes (Figure S4). After 16 h of salt treatment, Sodium-green fluorescence increased in root cells of control plants (Figure 5). Interestingly, in L3 and L5 cells from *SlSNAP33.2* overexpressing plants, the signal increase was more than twice the one of control plant cells (Figure 5B). Importantly, in control plants, the higher intensity of the probe signal was observed in epidermis cells and to a lesser extent in cortical cells. In contrast, in both L3 and L5 lines, the signal was clear and homogenous in both cell types, marking a significant difference between control and overexpressing plant cells. These observations suggest that sodium compartmentalization is directly related to the increased rate of endocytosis resulting in salt tolerance of plants overexpressing *SlSNAP33.2*.

## 3. Discussion

Tomato (*S. lycopersicum*) is one of the main food crops worldwide. However, its growth and associated productivity are strongly reduced under salt stress [31]. Tomato plants exhibit various physiological and biochemical responses to salt stress. One of the most important is the mobilization of phospholipids and proteins via exocytosis and endocytosis that allows the maintenance of membrane integrity. SNAP33 has a fundamental role in the fusion of vesicles with the plasma membrane during the exocytosis process [9], but its role in salt stress tolerance and associated vesicular trafficking has not been demonstrated in tomato plants. In previous work, we evidenced its induction during salt stress [18]. In this work, we found that the overexpression of *SNAP33.2* conferred salt tolerance to tomato plants. This increased resistance was related to the reduction in salt-induced oxidative stress on the one hand and to the enhancement of endocytic trafficking and the vacuolar compartmentalization capacity of the Na^+^ in roots on the other hand.

We confirmed the subcellular localization of SlSNAP33.2 suggested by [18], using a transient expression method in *A. thaliana* seedlings roots expressing different subcellular genetic fluorescent markers. The results showed that *Sl*SNAP33.2 is a Qbc protein located on the plasma membrane (Figure 1), consistent with what was demonstrated for the *At*SNAP33 protein and its homolog *Gs*SNAP33 [19,32]. Furthermore, this localization suggests that *Sl*SNAP33.2 has similar functions to *At*SNAP33 in the exocytosis related to cell division and the innate immune response [1,32].

Our study reveals that the overexpression of *SlSNAP33.2* conferred enhanced tolerance to salt stress and can improve tomato plant performance. The overexpressing lines we generated were less affected by salt stress than WT, displaying higher RWC, a higher content of total chlorophyll (chlorophyll A and chlorophyll B), observations that could be related to the greater turgor, and weaker sensitivity to leaves chlorosis during salt stress (Figure 2A,B,E–G). Moreover, the improved PI and Fv/Fm contributed to the salinity tolerance of *SlSNAP33.2*-overexpressing tomato plants, demonstrating an improved photosynthetic rate (Figure 2C,D). Environmental stress such as salinity results in oxidative stress, generating tissue damage, disrupting photosynthesis, and cell membrane damage [33]. Accordingly, the lower content of MDA in the L3 and L5 lines (Figure 2H) corroborates the increased tolerance to the high salinity of the transgenic tomato plants, suggesting that there is a lower membrane lipid peroxidation and reduced oxidative damage in response to salt stress. Other genes belonging to the SNARE and SNARE-like families that are induced under conditions of abiotic stress, *OsSYP71* from *Oryza sativa* and *SbSLSP* from *Salicornia brachiata*, respectively, have shown an enhanced oxidative stress tolerance in plants that overexpress these genes [34,35]. Conclusively all these findings would contribute to achieving enhanced stress tolerance in tomato plants subjected to high salinity conditions by maintaining the photosynthetic rate, a lower water loss, and less damage to cell membranes.

Our data showed that roots of tomato lines that overexpress *SlSNAP33.2* have a higher level of vesicle trafficking and endocytosis than wild-type during salt stress (Figure 3). These results correlate with observations in plant lines that constitutively overexpress elements that regulate the vesicular trafficking, such as *AtRabG3e* [30], *APYRASE2* from *Populus euphratica* [36], or *SchRabGDI1* from *S. chilense* [37], in *A. thaliana*, or *OsRab7* in *O. sativa* [38]. The increase in FM4-64 uptake in transgenic lines is also consistent with the evidence associated with increased endocytosis of proteins involved with water movement and ROS production, such as aquaporins and NADPH oxidases [2,21]. Note that FM4-64 has been shown to be an indicator of clathrin-mediated endocytosis [39], which accompanies PIP2 aquaporins when they are endocytosed by saline stress [40]. Additionally, reports show that members of the PIP2 family require SNARE SYP121 for their adequate intracellular trafficking [41], suggesting that its partner, SNAP33, could have potential involvement in the endocytic movement of these proteins. As a whole, the results indicate that the increase in endocytosis is triggered by the overexpression of *SlSNAP33.2*, and they are closely related to the increase in tolerance to saline stress in tomato plants.

The overexpression of *SlSNAP33.2* in tomato plants caused a significant reduction in H_2_O_2_, detected with the DCF indicator (Figure 4). Similar effects have also been evidenced in *A. thaliana* overexpressing *AtRabG3e* or *SchRabGDI1* [31,37]. In this context, [31] reported that the increase in endocytosis in *A. thaliana* roots allowed the reduction in the ROS induced by saline stress through the internalization of plasma membrane RbohD NADPH oxidases [42], consistent with the reduction in H_2_O_2_ we observed in tomato roots. Interestingly, the reduction in ROS and the increase in vacuolar sodium concentrations were closely related. It has been reported that the delivery of ROS to the vacuole regulates the activity of channels and pumps on the tonoplast, reducing both vacuole acidity and Na^+^ compartmentalization capacity [43,44]. In this respect, our results show an increase in sodium sequestration in the vacuole (Figure 5) associated with lower ROS accumulation, consistent with the observations in *A. thaliana* overexpressing *AtSFT12* [45].

The overexpression of *SlSNAP33.2* accelerated endocytosis in tomato plant roots. This is the first work that presents evidence of the role of SlSNAP33.2 in this pathway. However, to elucidate the mechanisms involved, more studies are required. Different reports indicate that the SNAP25-type plant family has an NPF domain that could allow them to interact with proteins involved in endocytic trafficking, such as EHD1 [3]. Similarly, it has been confirmed that SNAP33 interacts with VAMP721/722 SNARE, which has recently been associated with the endocytic pathway [46,47,48]. These antecedents suggest that the increase in tolerance to salt stress in transgenic tomato lines could involve other elements of the endocytic pathway, and the co-expression of *SNAP33.2* and *VAMP721* could be an alternative to improve this trait. The role of SNAP25-type proteins in exocytosis during abiotic stress is well studied. However, both secretory vesicular trafficking leading to exocytosis and internalization via endocytosis is essential to maintain cell homeostasis and membrane composition during abiotic stress [2]. SNAREs are key regulators of vesicular trafficking as they mediate membrane fusion between vesicles and target membranes, which determines the delivery of cargoes at specific cellular locations [46]. Together with the increase in endocytosis, the overexpressing plants showed greater compartmentalization of sodium in the vacuoles and a reduced ROS production in the root cells, being tolerant to this abiotic stress. Considering these results, it is possible that the overexpression of SlSNAP33.2 causes a disruption of the vesicular traffic of the exocytic pathway, increasing the deposition on the plasma membrane of specific phospholipids that improve membrane stability and facilitate the formation of microdomains [2,46] that cause the assembly in clusters of NADPH oxidase RbohD [49]. This favors the cellular internalization of RbohD through endocytosis, reducing the production of intracellular ROS [42] and generating a lower content of MDA in transgenic tomato plants. Induction of endocytosis would also cause the formation of endosomes containing sodium ions, which are subsequently accumulated in the vacuoles through the vesicular traffic of the endocytic pathway [37]. The accumulation of sodium in the vacuoles favors the K^+^/Na^+^ ratio in the cytoplasm of the root cells [50], reducing the sodium content that is transferred to the shoots. This decreases the osmotic effect of saline stress and also reduces the toxic effects of sodium, which is reflected in the fact that transgenic plants are less damaged by saline stress and show more favorable results in the physiological and biochemical parameters compared to wild type plants.

## 4. Materials and Methods

### 4.1. Subcellular Localization

The *SlSNAP33.2* CDS was amplified, as described in Section 4.5, and cloned into the pAM1 vector to obtain a fusion of the GFP gene to the 3’ end of the *SlSNAP33.2*. The resulting plasmid was used to transiently transform *A. thaliana* lines that express individually the subcellular markers RabG3f-mCherry, CS781670; RabF2a-mCherry, CS781672; VTI12-mCherry, CS781675; PIP1; 4-mCherry, CS781687; VAMP711- mCherry, CS781673; and SYP32-mCherry, CS781677 [28]. The described AGROBEST method was used [29]. Briefly, *A. tumefaciens* GV3101 transformed with the SlSNAP33-GFP construction was grown over solid 523 medium (Hydrolyzed casein 8 g/L, MgO_4_S 0.0358 g/L, KH_2_PO_4_ 2 g/L, Sucrose 10 g/L, Yeast extract 4 g/L, Agar 8 g/L, pH 6.9) supplemented with 50 mg/L of kanamycin and 5 mL of 24 h liquid culture. Then, bacteria were resuspended in 5 mL of AB-MES medium (K_2_HPO_4_ 2.99 g/L, NaH_2_PO_4_ 0.99 g/L, NH_4_Cl 1 g/L, KCl 1.5 g/L, MgSO_4_ 1.5 g/L, CaCl_2_ 0.14 g/L, FeSO_4_ 0.28 g/L, glucose 2 g/L, MES 10.66 g/L, and pH 5.5) supplemented with 200 µM acetosyringone and incubated for 16 h at 28 °C. Four day-old Arabidopsis seedlings were submerged in the bacterial suspension (OD_600_ = 0.02). After three days, seedlings were transferred to fresh liquid MS medium containing 100 µM timentin for two days. Roots were visualized upon a confocal microscope. GFP and mCherry were excited with lasers at 488 and 561 nm, respectively.

### 4.2. Plant Material and Saline Stress Assay

Wild type and transgenic tomato of *S. lycopersicum* cv. Moneymaker were grown in plastic pots with perlite, vermiculite and peat (1:1:1). Pots were watered once a week with a nutrient solution of 1.1 g/L of Murashige and Skoog (MS) medium [51] and grown in chambers with a photoperiod of 16 h of light and 8 h of darkness at 25 °C. For salt stress treatment, two overexpressor lines (L3 and L5) and WT of tomato plants of 10 to 11-week-old were irrigated with 200 mL of a 300 mM NaCl solution, every 3 days, for a period of 25 days, using [52] as a reference.

### 4.3. Physiological Parameters

Relative water content (RWC), Efficiency of Photosystem II (PSII), Performance Index (PI), Chlorophyll A, B and total, and MDA were estimated in leaves of tomato at 0, 5, 15 and 25 days after starting the salt treatment. At each time, 3 biological replicates and 3 technical replicates were evaluated.

#### 4.3.1. RWC

The fresh weight (FW), turgid weight (TW), and the dry weight (DW) of 6 leaves of the same size and location in the plant were measured. Leaves were initially weighed (FW) and then incubated in distilled water for 24 h at room temperature in darkness overnight. After this time, the excess water was removed with a piece of absorbent paper, and the tissue was weighed (TW). Finally, the samples were dried at 60 °C for 48 h (DW). RWC was calculated using the equation: RWC = [(FW − DW)/(TW − DW)] × 100 [53,54].

#### 4.3.2. Efficiency of PSII and PI

The efficiency of the PSII and PI were obtained with a hand-held Pocket PEA fluorimeter (Hansatech, King’s Lynn, Norfolk, England) following the procedure described by Giorio (2011) [55].

#### 4.3.3. Chlorophyll Content

To determine the chlorophyll content, leaf discs (0.5 cm^2^) were ground with 80% acetone (*v/v*), and the extract was centrifuged [52]. Then, the absorbance of the supernatant was determined by using the NanoQuant spectrophotometer (UV-160A, Kyoto, Japan). Chlorophyll concentration was estimated using the method described by Lichtenthaler and Wellburn (1983) [56].

#### 4.3.4. Lipid Peroxidation

Malondialdehyde (MDA) generated by fatty acid lipoperoxidation, an indicator of membrane lipid oxidation product of oxidative stress, was determined according to the modified protocol of Dionisio-Sese and Tobita (1998) [57]. Leaf tissue (0.5 g) was ground in 5 mL of 20% trichloroacetic acid (TCA). The supernatant was obtained by 5 min centrifugation at 5000× *g*. One mL of supernatant was extracted with thiobarbituric acid (TBA) at 0.5% (*m*/*v*) previously dissolved in TCA at 20% (*m*/*v*). The mixture was incubated at 95 °C for 25 min, cooled, and centrifuged (5000× *g*) for 10 min. The absorbance of the supernatant was measured at 532 and 600 nm, and the MDA was determined following the procedure described by Orellana et al. (2010) [52].

### 4.4. RNA Isolation and cDNA Synthesis

Total RNA was extracted from about 100 mg of leaves tissue of tomato plants using the commercial SV Total RNA Isolation System kit (Promega, Madison, WI, USA). In order to eliminate the remaining DNA, each sample was treated with DNAse I (Ambion^®^ TURBO DNA-free™). The RNA integrity was verified in agarose gels at 1% (*w/v*) (Figure S5). The concentration and purity of the RNA were estimated at 260/280 nm absorbance using a NanoDrop ND-1000 (NanoDrop Technologies, Wilmington, NC, USA).

Synthesis of the first strand of cDNA was performed with 1 to 2 µg of total DNA-free RNA and the First Strand cDNA Synthesis kit system (Fermentas). The reactions were carried in a final volume of 20 μL, containing: 10 μL of RNA (1 to 2 μg), 1 μL oligo (dT), 4 μL reaction buffer (5×), 1 μL of ribonuclease inhibitor RiboLock, 2 μL of 10 mM dNTPs, and 2 μL of reverse transcriptase MMuLV. The reaction was incubated at 37 °C for 60 min, and transferred to 70 °C for 5 min to stop the reaction. cDNAs were stored at −20 °C for later use.

### 4.5. Isolation of the SlSNAP33.2 Gene

To amplify the coding sequence of *SlSNAP33.2* gene (Solyc06g069570.3.1), primers SNAP33_Fw: 5’-TCTAGAATGCATGGTCTTAAGAAGTCTCCTT-3 ‘and SNAP33_Rv: 5’-CCCGGGTTACTTTCCAAGCAAGCGCC-’3 were designed. These primers contain XmaI and XbaI restriction enzyme sites, respectively, for further cloning in a plant vector. The PCR reaction was performed in a final volume of 25 μL using the high-fidelity Platinum Taq DNA Polymerase following the manufacturer’s instructions (Invitrogen, California, USA). The thermal profile of the PCR was: 95 °C for 5 min, 35 cycles of 95 °C for 45 s, 55 °C for 60 s, 72 °C for 60 s, and final extension for 7 min at 72 °C. The PCR product was separated by 1% *w/v* agarose gel electrophoresis (1X TAE Buffer). A single band with the predicted size was obtained and purified using the EZNA Gel Extraction system (Omega Bio-Tek, Georgia, USA). The amplified fragment was cloned in the pGEMT-easy vector following the standard protocol (Promega, Wisconsin, USA). The cloned PCR product was sequenced to confirm the fidelity of the isolated gene (MACROGEN, http://www.macrogen.com (accessed on 6 June 2021)).

### 4.6. Generation of Tomato Transgenic Plants

The full length of the SlSNAP33.2 CDS was replaced for the *GUS* sequence in the pBI121 vector by using the XmaI and XbaI sites on the SNAP33_Fw and SNAP33_Rv primers, generating after all the 35S::SlSNAP33 cassette. The new construct was introduced by a chemical transformation in *Agrobacterium tumefaciens* GV3101 pmp 90 strains [58]. The transformed bacteria were grown in solid YM medium (0.04% *w*/*v* yeast extract, 1% *w/v* mannitol, 1.7 mM NaCl, 0.8 mM MgSO_4_ • 7H_2_O, 2.2 mM K_2_HPO_4_, and 1.5% *w/v* agar) with rifampicin 100 (mg/L), gentamicin (25 mg/L), and kanamycin (50 mg/L). After 48 h at 28 °C, colonies were analyzed by PCR. Recombinant clones were used to transform cotyledon tomato explants, according to the method of Fillati et al. (1987) [59]. Transgenic plants were selected with kanamycin (50 mg/L) and micropropagated continuously to maintain a stock of plant biomass. The T0 transgenic lines were analyzed by PCR and qPCR. Several transgenic lines carrying the 35S::SlSNAP33 cassette and expressing the *SlSNAP33.2* were identified. The tomato plants transformed with the empty vector were used as a control (vector).

### 4.7. SlSNAP33.2 Expression on Tomato Transgenic Plants

The transgene transcript levels were determined by qRT-PCR using the SNAP33q_Fw: 5’-GACTGAACTCAGCACCCAGAG-3’ and SNAP33q_Rv: 5’-ATTGATTGTCGAGTTTGTGGC-3’ primers. The analysis was performed with the “Brilliant SYBR Green Master Mix’’ kit following the manufacturer’s instructions (Stratagene, California, USA) (Figure S6). Each analysis was performed with three biological replicates with three technical replicates each. The reaction was carried in a final volume of 20 μL, which contained: 10 μL of Master MIX, 0.5 μL of each primer (250 nM), 1 μL of cDNA (25 ng total), and nuclease-free water. The conditions of the qPCR were as follows: 95 ° C for 10 min, followed by 40 cycles of 95 °C for 15 s, 62 °C for 20 s, and 72 °C for 20 s. The 2^−ΔΔCt^ method was applied to calculate the rate of change of the transcript levels of the transgene [60,61].

### 4.8. Evaluation of Subcellular Phenotypes

#### 4.8.1. Endocytic Rate in Root Cells

Five-day-old tomato seedlings were incubated with the lipophilic indicator FM4-64, 5 μM, at 4 °C, for 15 min (Excitation 515 and emission range of 640–734 nm, Molecular Probes, Eugene, OR) [62]. Samples were washed in a solution of liquid MS medium at 25 °C and carefully placed on a slide (time 0). Seedlings were observed under a confocal microscope, and images were obtained at 5, 15, and 30 min. Then, to determine the effect of saline stress on the endocytic rate, a group of seedlings was treated for 20 min with NaCl 200 mM on liquid MS medium and subsequently incubated with FM4-64 5 μM, in darkness and 4 °C, for 15 min. The endocytic rate was calculated following the procedure described by Baral et al. (2015) [63]

#### 4.8.2. Determination of H_2_O_2_ in Roots

The protocol was adapted from Leshem et al. (2006) [42]. Tomato seedlings were exposed for 40 min to a liquid MS medium supplemented with NaCl 200 mM. Then seedlings were stained for 20 min with the tracer H_2_DCFDA 10 mM (2’,7’-dichlorodihydrofluorescein diacetate, Thermo Fisher Scientific, Waltham, MA, USA). Afterward, the seedlings were incubated for 20 min with FM4-64 5 μM. The fluorescence intensity of the H_2_DCFDA product, DCF, was evaluated (excitation 492 nm and emission 517 nm)

#### 4.8.3. Determination of the Presence of Vacuolar Na^+^ in Roots

To determine the presence of Na^+^ into the vacuole, tomato seedlings were treated according to a methodology adapted from Tarte et al. (2015) [45]. Seedlings were incubated for 16 h in liquid MS medium supplemented with NaCl 200 mM and then transferred for 2 h to MS medium containing 5 μM of Sodium Green (Thermo Fisher Scientific, Waltham, MA, USA) and Pluronic F-127 20% *w/v* (Thermo Fisher Scientific, Waltham, MA, USA). Sodium Green was detected by confocal microscopy (excitation 507 nm and emission 532 nm).

### 4.9. Microscopy

All images were obtained using a Zeiss LSM 700 laser scanning confocal microscope, 40× immersion objective, and 0.8× or 4× digital zoom. The images were analyzed using ImageJ software (http://rsb.info.nih.gov/ij/ (accessed on 6 June 2021)). For endocytosis, about 25 cells from three different roots were analyzed at each treatment and time point for each genotype. For the determination of H_2_O_2_ and Na^+^, at least 5 roots from 4 to 5 different plants for each genotype were analyzed. Analysis was repeated three times.

### 4.10. Statistics

To interpret the results, statistical analyses were performed in R (version 1.7). Statistical significance was analyzed by one-way ANOVA followed by the post-hoc Tukey HSD test; *p* < 0.05 was considered significant.

## 5. Conclusions

The *Solanum lycopersicum* gene *SlSNAP33*.2 encodes a protein that was accumulated at the plasma membrane in root cells of *A. thaliana*. The overexpression of *SlSNAP33.2* resulted in a higher rate of endocytosis of *Solanum lycopersicum* plants. Furthermore, the higher level of *SlSNAP33.2* gene product diminished the toxicity of salt stress by the accumulation of sodium into the vacuoles and a reduction in intracellular ROS on root cells. Consistently, *S. lycopersicum* overexpressing *SlSNAP33.2* displayed a better performance when faced with salt stress conditions indicating that the high level of this protein confers resistance to such environmental cues in the whole organism.

## Data Availability

Not applicable.

## Supplementary Materials

The following are available online at https://www.mdpi.com/article/10.3390/plants10071322/s1, Figure S1: Subcellular distribution of GFP markers, Figure S2: Relative expression of *SlSNAP33.2* in transgenic lines. Figure S3: Detection of H_2_O_2_ in tomato roots under control conditions. Figure S4: Detection of Na^+^ in tomato roots under control conditions. Figure S5: RNA integrity in agarose gel 1% *w/v*. Figure S6: Melting Curve Analysis.
