# Supplementary figures and images for: The Exocytosis Associated SNAP25-Type Protein, SlSNAP33, Increases Salt Stress Tolerance by Modulating Endocytosis in Tomato
Source: Plants (Basel). 2021 Jun 29;10(7):1322. doi: 10.3390/plants10071322 (PMC8309203; doi:10.3390/plants10071322)

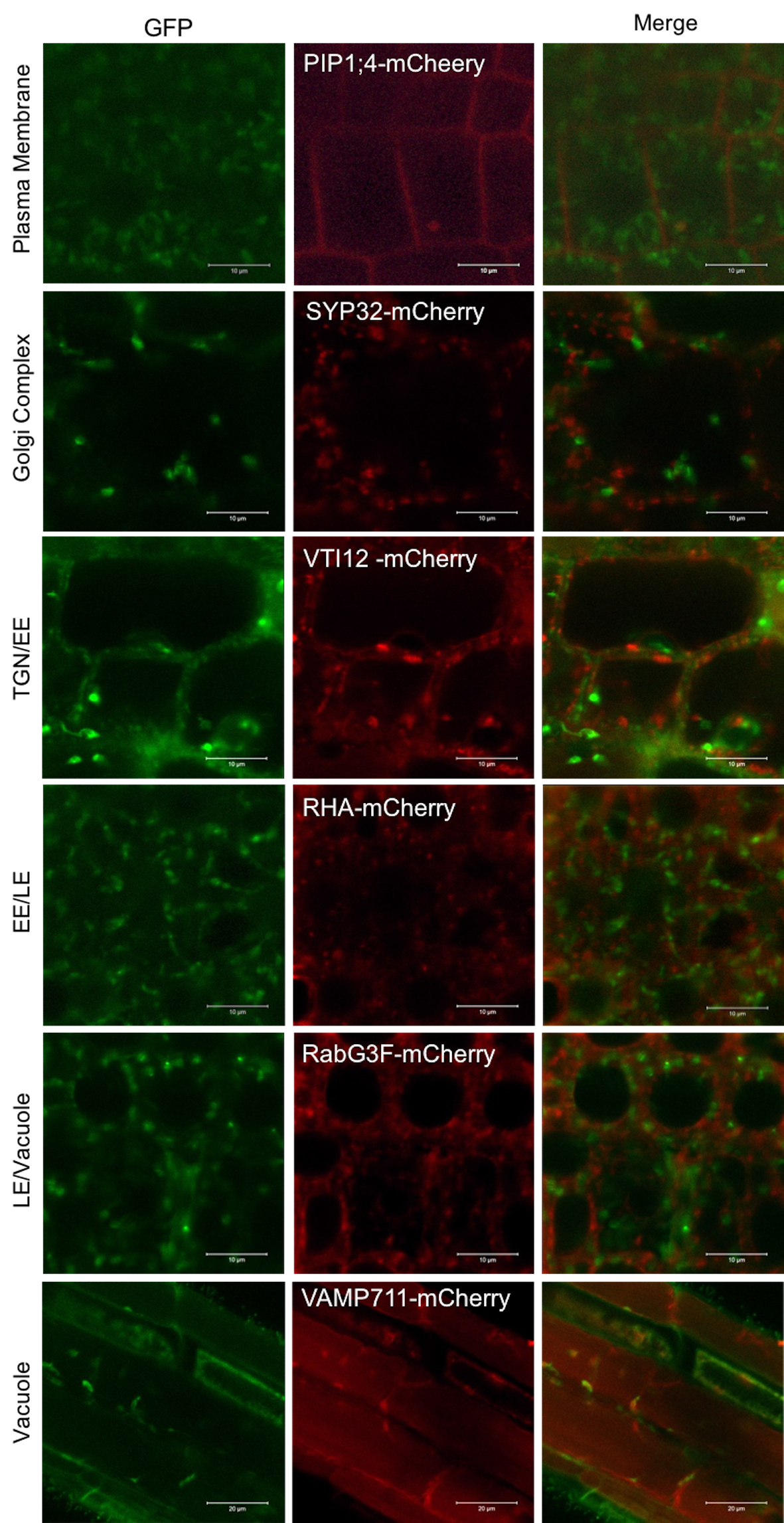

Supplement: Supplementary file 1 [file plants-10-01322-s001.zip › Figure S1.png]

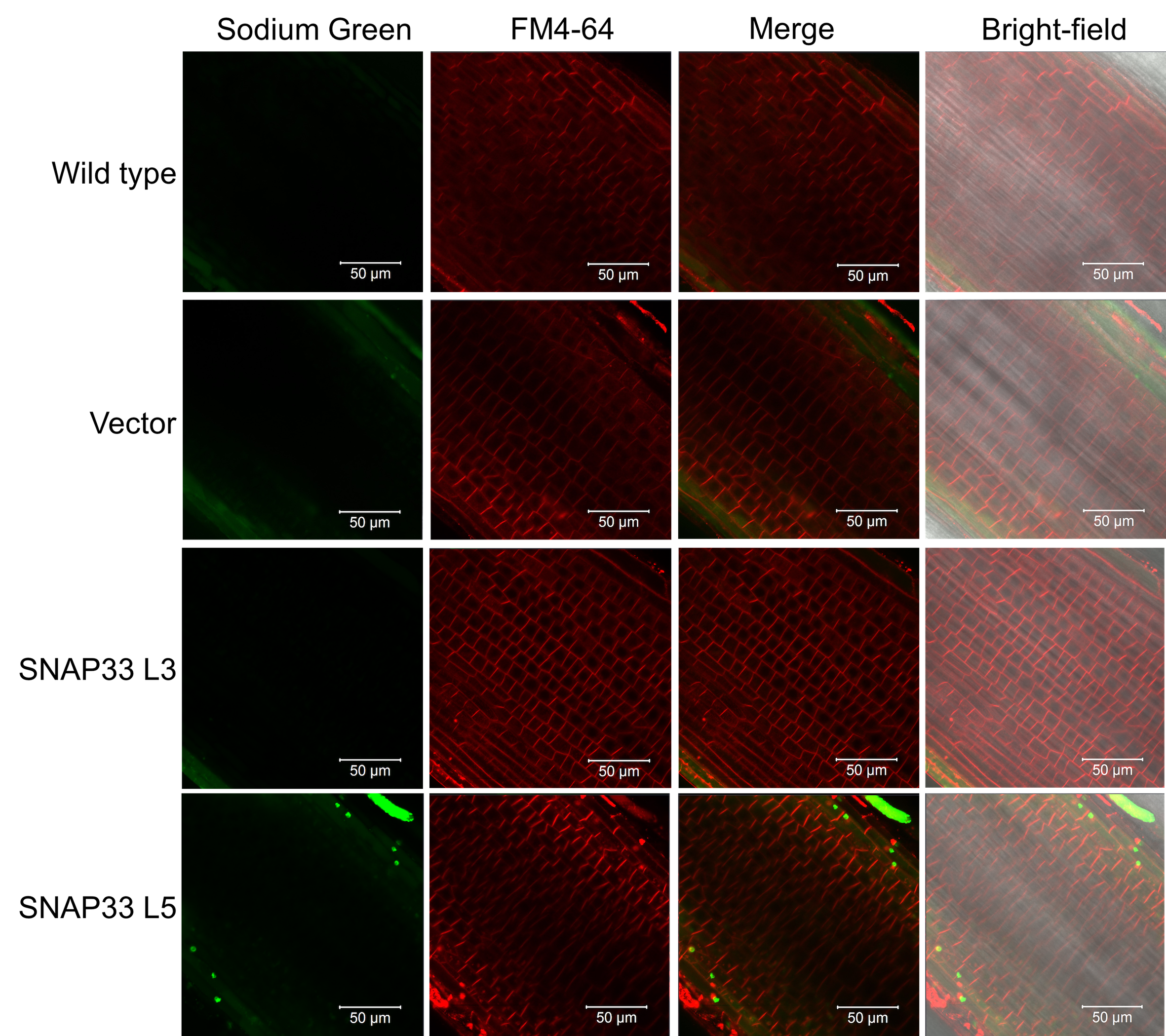

Supplement: Supplementary file 1 [file plants-10-01322-s001.zip › Figure S2.png]

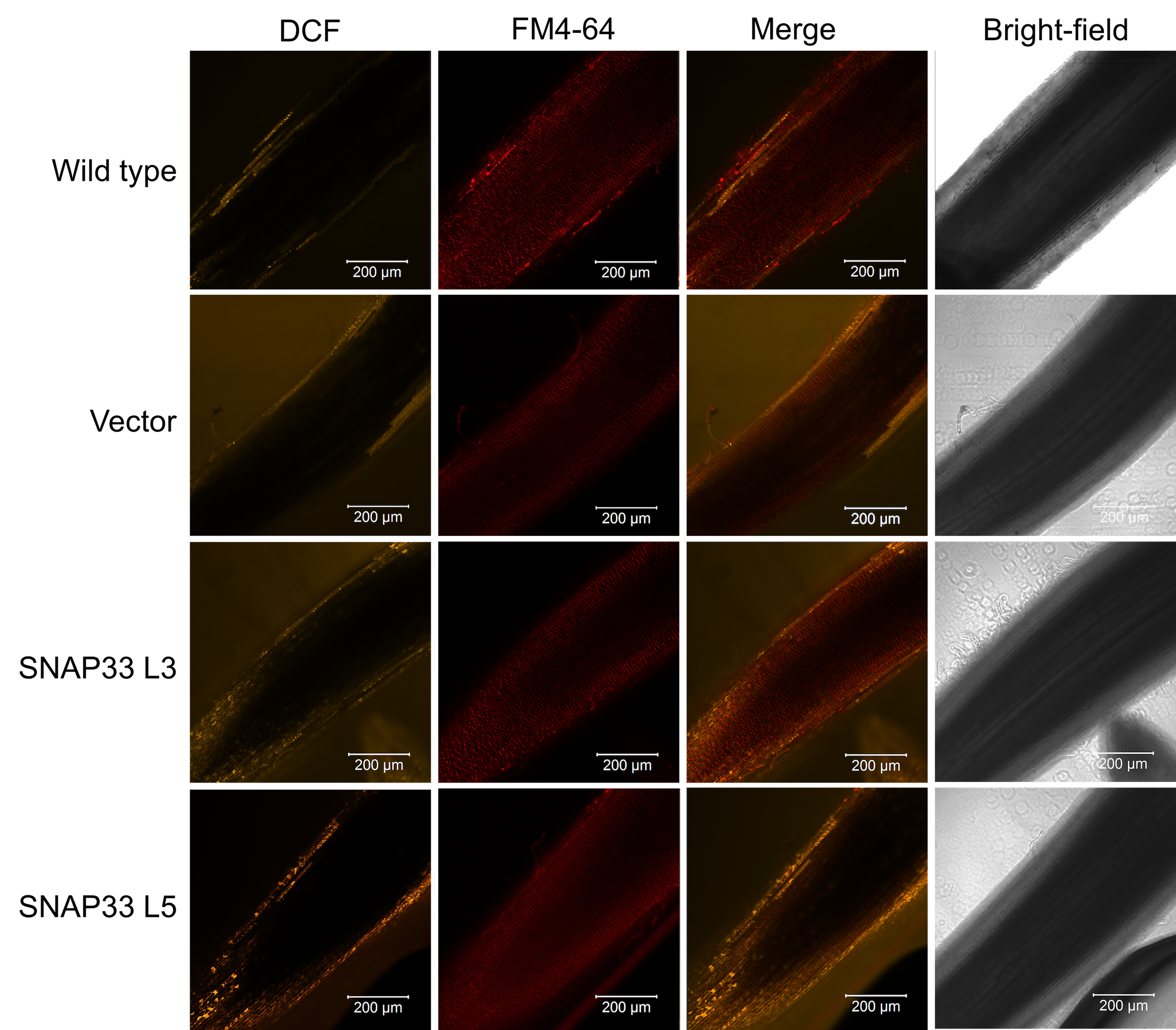

Supplement: Supplementary file 1 [file plants-10-01322-s001.zip › Figure S3.png]

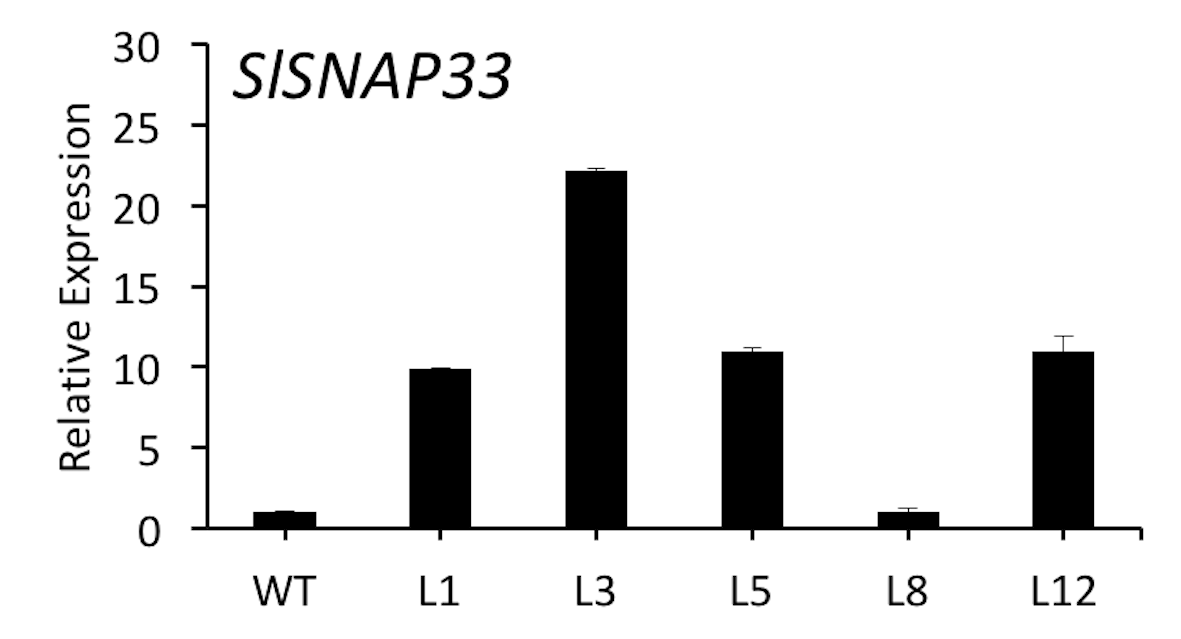

Supplement: Supplementary file 1 [file plants-10-01322-s001.zip › Figure S4.png]

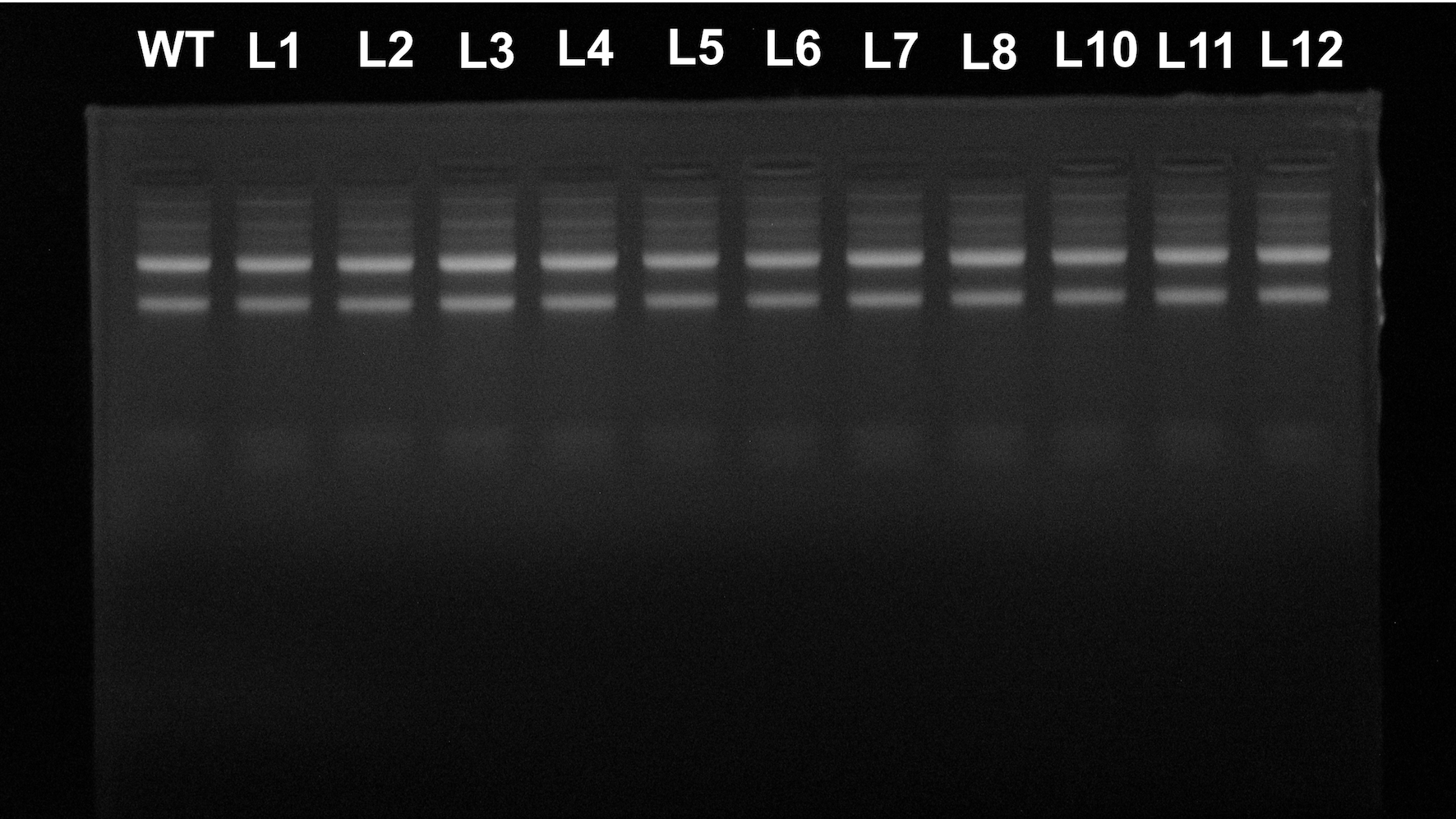

Supplement: Supplementary file 1 [file plants-10-01322-s001.zip › Figure S5.png]

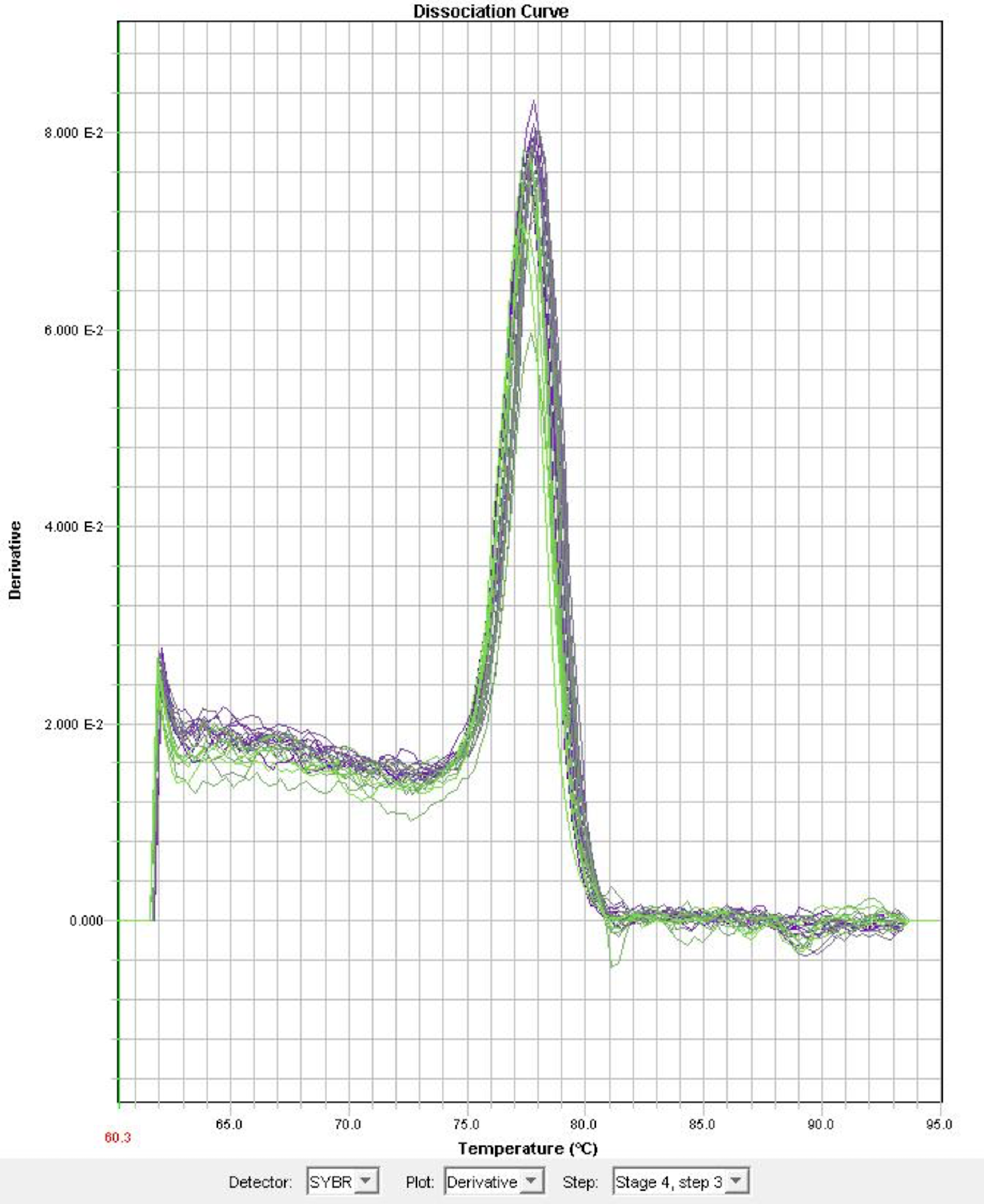

Supplement: Supplementary file 1 [file plants-10-01322-s001.zip › Figure S6.png]
